# The Temporal Rating of Emergency Non-Technical skills (TRENT) index for self and others: psychometric properties and emotional responses

**DOI:** 10.1186/s12909-014-0240-y

**Published:** 2014-11-28

**Authors:** Eamonn Ferguson, Andy Buttery, Giulia Miles, Christina Tatalia, David D Clarke, Adam J Lonsdale, Bryn Baxendale, Claire Lawrence

**Affiliations:** Personality and Social Psychology (PSPH) group, School of Psychology, University of Nottingham, Nottingham, NG7 2RD UK; Trent Simulation and Clinical Skills Centre, Queen’s Medical Centre, Nottingham University Hospitals NHS Trust, Nottingham, UK; Department of Psychology, Oxford Brookes University (Formally PSPH group), Oxford, UK

**Keywords:** Self-assessment, Peer-assessment, Performance, Stress, Emotions, Reliability, Inter-rater

## Abstract

**Background:**

To enhance the non-technical skills (NTS) assessment literature by developing a reliable and valid peer and self-assessment tool for NTS in a simulated ward setting to include emotional reactions: the Temporal Rating of Emergency Non-Technical skills (TRENT) Index. The paper aims to document (1) the psychometric properties of the TRENT index (e.g., reliability, idiosyncrasy biases) and (2) its validity in terms of performance-emotional associations in the high fidelity simulated ward environment.

**Methods:**

Two samples of doctors (Ns =150 & 90) taking part in emergency simulations provided both self and peer-assessment of NTS, with the second sample also providing self-assessments of mood. The psychometric properties of the TRENT were explored for self- and peer-assessment, and pre- and post-simulation environment mood was used to assess validity.

**Results:**

A psychometrically reliable and valid 5-factor assessment of NTS was developed. While there was evidence for both intra-rater and inter-rater reliability, inter-rater idiosyncrasy was also observed. Self-rated, but not peer-rated, negative performance was positively associated with post simulation negative mood.

**Conclusion:**

These are the first results that pertain to inter-, intra-rater reliability as well as idiosyncratic biases in NTS assessment and the first to show that simulator performance can influence mood after assessment. Potential clinical carry-over effects of mood are discussed.

**Electronic supplementary material:**

The online version of this article (doi:10.1186/s12909-014-0240-y) contains supplementary material, which is available to authorized users.

## Background

Simulation is increasingly used to enhance the development of clinical (technical) and non-technical skills (NTS) in healthcare professionals [1]. Non-technical skills refer to cognitive, social, interpersonal and emotional skills, rather than the technical aspects of clinical care (e.g. giving an injection, inserting a urinary catheter), that support effective teamwork and interaction and may be considered generic to life as well as specific job roles [2-4]. While poor NTS have been identified as potentially detrimental to safe and effective care [1], it is only recently that they have started to feature within undergraduate or postgraduate curricula. Observing and providing effective feedback on NTS in postgraduate specialty trainees has proven slow to implement, as existing assessment instruments have tended to be role-specific and use a single source of feedback: peer-assessments [5]. This contrasts with the wider literature on OSCE’s (Observed Structured Clinical Exams) and workplace-based assessments of clinical skills which recommend multi-source feedback (e.g., both self and peer) [6-8]. Students also perceive multi-source feedback as fairer [9].

Therefore, a single instrument that combines validated and psychometrically reliable peer- and self-assessment of NTS would be a useful addition to the medical education literature. The main aim of this paper is to report the development of such an instrument that fills this gap in the literature by including both self *and* peer assessments of NTS within a single reliable and valid instrument, for use within a simulated ward environment for Foundation Programme trainee doctors^a^. This instrument is termed the Temporal Rating of Emergency Non-Technical skills (TRENT) index. This study is set within the framework of an on-going project assessing the impact of NTS in the simulated environment on future clinical practice.

### Assessing Non-Technical Skills (NTS): why self and observer evaluations are both needed

Why do we need both self and peer-ratings to be measured simultaneously with the same items? Simply, peer reports are believed to be more reliable, and less biased by social desirability (the over-estimation of good aspects of one’s own behaviour and under-estimation of bad aspects: [10]) than self-ratings and, as such, are considered the gold standard for assessment [11]. However, peer-reports are not immune to biases themselves. Indeed peer-assessment suffers from Halo and Horn Effects (attributing additional positive/negative traits or behaviour on the basis of the evidence of an existing positive /negative behaviour or trait), the ‘Mum Effect’ (the unwillingness to give ‘negative’ evaluation or feedback) and more physically attractive candidates being preferentially rated [12].

Thus, accurate identification of sources of unreliability in assessment requires both peer-and self-observation [8]. Peer-assessments enable us to calculate both *inter*-rater (the degree of agreement between two or more raters when they both evaluate the same individual) and *intra*-rater reliability (the degree of stability of evaluations from one rater using the same rating scale at two time points: sometimes called the stability coefficient) [13]. Viswesvaran et al. [13] showed that the difference between the inter-rater reliability and the stability coefficient gives an estimate of variance due to rater *idiosyncrasy* (preference for certain behaviours, attention, mood etc). If much of the variance in peer-assessment reflects rater idiosyncrasy, then less confidence can be given to those assessments. This paper is the first to report rater *idiosyncrasy* for NTS in simulated medical environments.

### Validity

#### Self-peer associations

Within the personality trait literature, the association of self- and peer-assessments is part of the validation process [10]. While this is valid within the context of stable traits, both self- and peer-assessments are likely to be less stable in a domain like NTS. This study, therefore, will be the first to explore the degree of correlation between self- and peer-assessments of NTS.

### Emotion responses: the missing component of existing models of NTS in high fidelity simulation contexts

There are a number of omissions in current instruments assessing NTS (see Table 1). First and foremost the role of emotions is not typically included within existing NTS instruments (for an exception see [14]). This is despite emotions being part of models of medical competency [15,16]. Thus the second major aim of this paper is to extend the assessment of self- and peer-observed NTS to include salient emotional components.

**Table 1:** **Existing Models and Measures of NTS and the TRENT index Dimensions**

	**TRENT Dimensions**				
	**Introduces and interacts with the patient**	**Focuses on colleagues rather than self**	**Attends and reacts to the environment**	**Avoids taking the lead**	**Offers social support**
***Other Models***					
Fletcher et al. (2003) the ANTS [5]		Team Work	Task management, Situation awareness, Decision making		
Moorthy et al. (2005) [17]	Preparation	Communication and interaction	Vigilance/Situational awareness, Leadership		
Von Wyl et al. (2009) [18]		Task delegation, Team leader’s communication, Team member’s communication, Team work	Leadership, Team member’s individual responsibility		
Gale et al. (2010) [14]		Communication, Team working	Achievements, Situational awareness, Organization and planning	Working under pressure,	

*Note*. ANTS = Anaethetist’s Non-Technical Skills.

Emotions are an important aspect of all medical work both in terms of influencing the doctor’s own behaviour and the doctor’s response to other’s emotional reactions [19]. A doctor’s response to others’ emotional reactions is predicated on those emotional responses being observed. In ongoing interactions, people can use subtle facial and bodily cues to express emotions and other’s to encode them [20]. However, in the context of observing and recording behaviour in a simulated medical context (or indeed an actual live medical context) the pronounced behavioural patterns elicited by emotions are likely to be more easily observed and to form the basis of a more reliable recording of ongoing emotions [19]. In terms of a general peer-rating instrument, subtle facial cues are unlikely to be easily observed, and indeed these are often observed outside of conscious awareness [20]. As such, the TRENT index focuses on behavioural responses (e.g. withdrawal and approach) associated with anxiety as a specific behavioural response by the doctor and *social support* as a response to other’s emotions. Anxiety was chosen as a target emotion because (1) the key behaviours associated with anxiety (withdrawal and approach) are easy observable and (2) it is the emotion most likely to predominate in both evaluative and highly emotionally charged contexts [21]. It should be noted that anxiety, as well as supporting ‘withdrawal’ (flight) also has an ‘approach’ function [22,23]. That is, anxiety helps us to monitor our environment for danger and to approach (fight) the source of danger [23]. The items we use to assess responses to anxiety therefore, contain references to both behavioural withdrawal and approach. Similarly offering help to colleagues exhibiting distress is a dominant and intuitive choice, by the majority, when people see others in distress [24,25]. Theoretically, the literature distinguishes between emotional and practical social support and as such our items are designed to assess these two different aspects of social support [26,27]. Thus, the TRENT index aims to fill a gap in the literature by adding emotional components (e.g., observable responses to anxiety & offering support) to existing dimensions from other frameworks (e.g., building patient rapport).

Finally, anecdotally there is a temporal sequence to most medical encounters: the doctor meets the patient, introduces themselves and builds a rapport, followed by history taking, discussing the case and treatment plans with colleagues, progressing to monitoring, reacting to any clinical changes, seeking advice and ordering appropriate tests to confirm or refute the initial diagnosis. The doctor may become more or less anxious or uncertain about how to proceed at any point and support may be offered by, or to, colleagues. This temporal ordering has two implications. First, if peers are to observe their colleagues, then having evaluative items presented in a logical temporal order will make them easier to locate and use. Second, emotional responses are likely to emerge more strongly as the encounter progresses, thus emotional ratings should be towards the end of any set of evaluative ratings. Thus the temporal order of items is likely to be crucial to the assessment of NTS, and the TRENT index orders the behaviours in the index with respect to the temporal flow of a medical encounter. The TRENT index dimensions are shown in the top row of Table 1, which details how they relate to existing measures. The exact TRENT index items are detailed in Additional file 1.

### Validity of NTS emotional assessments: the role of doctors’ emotion and NTS performance ratings

As part of examining the validity of these newly included emotional components we explore their associations with mood assessed before and after being in a stressful simulated medical context [28,29].

Theoretical work indicates that assessments of risk and behaviour are influenced by emotions and mood [30]. The *‘risk-as-feelings’* hypotheses suggests, for example, that a person’s assessments of their behaviour and risk are influenced by their emotions and mood, suggesting that others’ assessment of the same behaviour will not necessarily take into account the emotions of the person being assessed [30]. Similarly the concept of a *‘interpersonal hot-cold empathy gap’* suggests that a person in one emotional state (e.g. the peer-observer in a cold emotional state) cannot assess how emotions will influence the behaviour of a person in a different emotional state (e.g. the assessed doctor in a hot emotional state) [31]. Together this reasoning suggests that for the doctor being assessed, their self-assessed performance during simulation should be associated with their reported mood pre- and post-simulation. However, the assessed doctor’s pre- and post-mood should not be associated or only weakly associated with their peer assessed performance (i.e. peer assessors may not account for the assessed doctor’s emotional state when judging them). This study is, to the authors’ knowledge, the first to assess the effects of the assessed doctor’s mood on the assessments of their NTS.

### Aims of the paper

This paper will report on the TRENT index of NTS, and will be the first to:

1. index both *self*- and *peer*-assessment of NTS,
2. assess inter-rater and intra-rater reliabilities and rater idiosyncrasy, and
3. explore the influence of doctors’ emotions on self- and peer-assessed performance.

### Pilot study

The following task analysis procedure was adopted to generate the initial item set for the TRENT index. This involved reviewing the types of behaviours exhibited in the simulated contexts in which the TRENT index was to be applied, interviews with key personnel and a review of existing measures and the theoretical literature [16]. Initially two chartered psychologists (EF, CL) reviewed (with permission) recordings of doctors’ performance in the high-fidelity simulated ward acute care situations at the Trent Simulation and Clinical Skills Centre (TSCSC) at the Queen’s Medical campus of Nottingham University Hospitals NHS Trust in Nottingham (http://www.nuh.nhs.uk/our-services/services/trent-simulation/) and attended two training days as observers. Based on these observations a series of key task parameters were identified (e.g., introductions, seeking advice and help, setting out a treatment plan). Following this these key themes were discussed with a team of experienced simulation trainers and faculty staff (BB, AB, GM). Following these discussions items were generated and piloted during 2–3 training days for comments and feedback. Items were then rewritten in further discussion with faculty staff. The theoretical literature on social support and anxiety along with key existing measures was reviewed (see Table 1). Based on this process 27 items were generated and grouped into the five dimensions in Table 1:

1. Introduces and interacts with the patient
2. Focuses on colleagues rather than self
3. Attends and reacts to the environment
4. Avoids taking the lead
5. Offers social support.

Responses for each item were coded:

- 0 = ‘not applicable’
- 1 = ‘not performed’
- 2 = behaviour was performed to a ‘limited extent’
- 3 = the behaviour was ‘definitely’ performed.

Twenty-six items were used in the final analyses^b^. These were completed in self- and peer-assessment forms by 150 F2 doctors, attending simulation training at the TSCSC. The ‘not applicable’ category showed a low frequency (6% across items) and so the items were rescaled to 0 – 2, changing 0 to represent a combination of ‘not applicable’ and ‘not performed’ the behaviour [32,33].

The simulation context consisted of a full day training course attended by F2 doctors. This used a high-fidelity manikin and environment to present a series of 8 clinical scenarios, which were performed in the same order on every course day. All course days were conducted at the TSCSC. Each scenario contains a different clinical and professional challenge and may involve a combination of the following: emergency situations, ethical/religious considerations, dealing with an upset or angry relative, and challenging a senior when necessary. Participants undertook the scenarios in pairs, with one doctor taking the lead and the other providing a supportive role with their peer observer watching through a one way mirror. The other non-participating doctors (who are not peer observing) also watched each scenario, as did faculty staff. This was followed by a post-simulation debrief.

**Figure 1:**
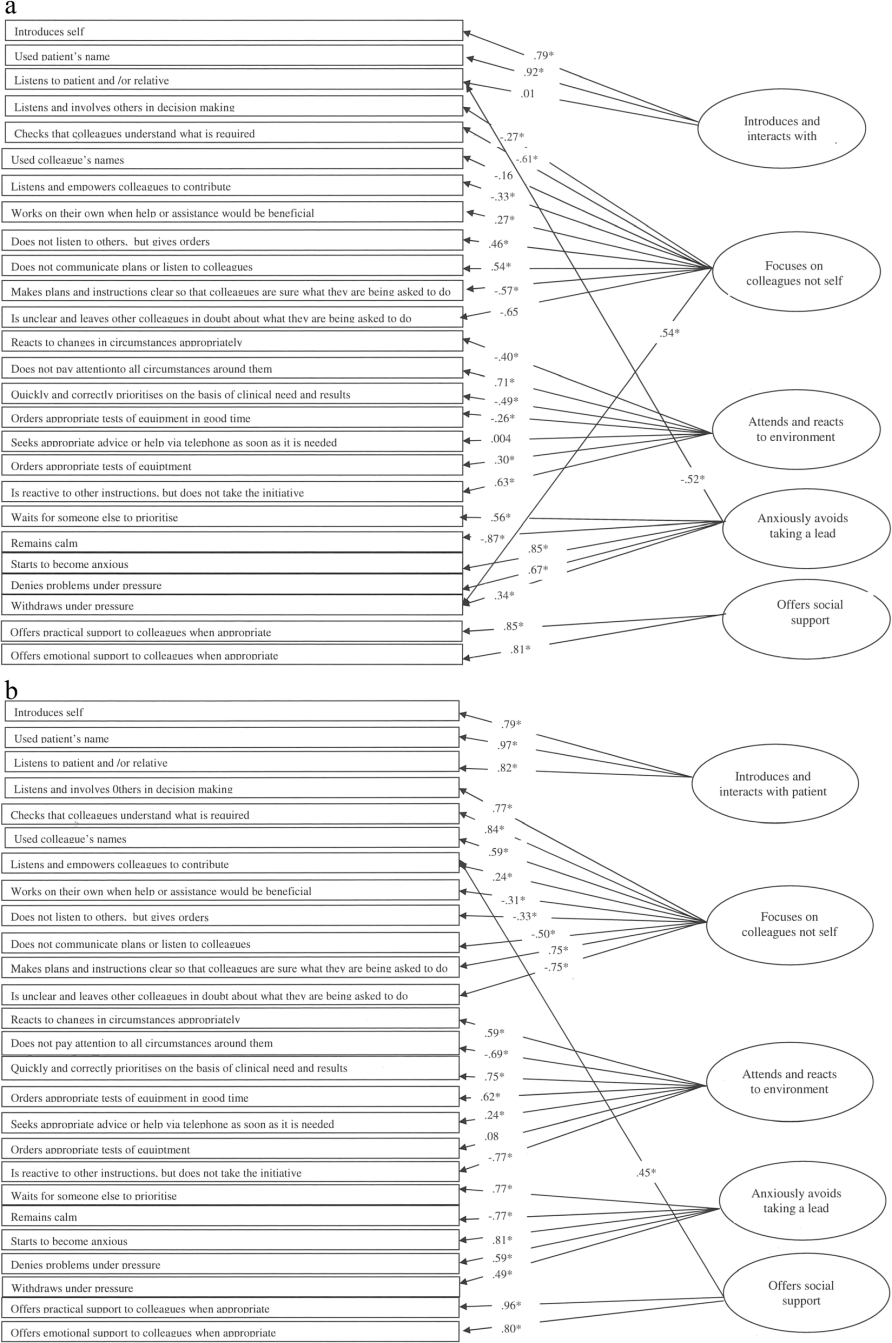
**Confirmatory Factor Analytic Models For Self-Reported (a) and Peer-Reported (b) Non-Technical Skills.** The figure details the items (rectangular boxes) and the latent factors (ellipses). The coefficients are standardized factors loading. *p < .05.

Confirmatory factor analysis in M*plus* 7 [34] using diagonally weighted least squares (WLSM) showed that the five dimension model had a good fit for the self-assessment when two addition loadings are specified after consulting modification indices (N =144: CFI = .90, RMSEA = .067, WRMR = .97)^c^. The fit of this model further improved if the three non-significant loadings were removed (CFI = .92, RMSEA = .065, WRMR = .94). For the peer-assessment the fit with one specified modification was also good (CFI = .92, RMSEA = .08, WRMR =1.0). These models are shown in Figures 1a and b^d^.

### Ethical approval

This study was approved as a service evaluation and gained ethical approval from the School of Psychology Ethics Committee, University of Nottingham. All participating doctors provided written informed consent to participate.

### Main study: assessment of rater biases and effects of mood

Based on the results of the pilot study, a revised version of the TRENT Index was developed to increase the number of items per behavioural domain and their conceptual clarity, remove the redundant non-applicable category and enhance the temporal order.

## Method

### Participants

Ninety F1 doctors participated (M age = 25.8 years, SD = 4.1: 51% male) who were on average 10 months into their current role when attending the simulation course day. This study is part of a larger study but here we only report on the psychometric properties of the TRENT index.

### Procedure

Doctors participated in the simulation in randomly assigned staggered pairs with one acting as the lead doctor. Each scenario contained a different clinical challenge and allocation to these scenarios was randomised across doctors. The lead doctor provided ratings of their emotions pre and post simulation^e^. The lead doctor rated their own performance prior to assessing their post emotions and was rated by two peers (in real time) simultaneously. Each doctor also rated two separate lead doctors. All doctors (usually 8) attending each day observed each scenario even if they were not acting as peer observers.

### Measures

#### Emotions

These were assessed using a short version of the UWIST MACL [35,36]. Participants indicated how each of 7 adjectives described how they felt at that moment, using a four-point Likert-type scale, from ‘definitely’ to ‘definitely not’ [36]. Items were scored to form 4 mood scales:

1. Hedonic Tone (Happy and Depressed (reversed) (higher scores indicated feeling happy): MIC _pre_ = .42, MIC _post_ = .42)
2. Tense Arousal (Relaxed (reversed) and Anxious (higher scores indicating feeling anxious): MIC _pre_ = .46, MIC _post_ = .46)
3. Energetic Arousal (Sluggish (reversed) and Active (higher scores indicating feeling active): MIC _pre_ = .33, MIC _post_ = .36)
4. Annoyed (higher scores indicating feeling annoyed).

For scales with fewer than 4 items the mean inter-item correlation (MIC) is the appropriate index for estimating (reliability range from 0.1 to 0.5) [37]. As such these scales were found to be reliable.

#### Revised TRENT index

Self and peer NTS assessments ratings were made using a revised TRENT Index consisting of 33 simplified single statements, presented in the predicted sequence of the unfolding clinical event and measured on a 3 point scale (0 = didn’t do it, 1 = did it a little and 2 = did it a lot) (see Additional file 1). Items were revised following the pilot study. For example the original item “Listens and involves others in decision making” assessed two behaviours “listening” and “involving” and was split into “Listens to colleagues” and “Involves colleagues in decision making”. Similarly for “Listens and empowers colleague to contribute” only the “Empowers colleagues to contribute” was retained as the “Listening” component was already assessed.

### Ethical approval

This study received NHS National Research Ethic Service (NRES) approval number 09/H0408/25 from Nottingham Research Ethics Committee 2. All participating doctors provided written informed consent to participate.

### Statistical analysis

Reliability of multi-item scales (more than 4 items) was assessed using Cronbach’s coefficient alpha where values equal to or greater than 0.70 indicates reliability. For scales with fewer than 4 items the MIC is reported [26]. The sample size was not sufficient to apply CFA procedures. However, as the CFA in the pilot study was a good fit, confirming the basic conceptual model, good internal reliability for each scale as well as theoretically consistent correlations between scales will be regarded to confirm conceptual validity of the TRENT Index.

## Results

### Internal reliability

The internal reliabilities of the 5 behavioural domains for the self- and the two peer-assessments are reported in Additional file 1. ‘Focuses on colleagues rather than self’, ‘Avoids taking the lead’, ‘Attends and reacts to the environment’ and ‘Offers social support’ were all consistently reliable. However, the reliability of ‘Introduces and interacts with the patient’ scale for Peer assessment 2 was unacceptable due to restricted variance, making the assessment of reliability statistically problematic (the reported frequency of the first three behaviours across the self and peer ratings ranged from 88 to 99%). Thus overall the TRENT index scales were reliable.

### Intra-rater reliability (Stability) & inter-rater reliability

With respect to intra-rater reliability, Table 2 shows that, apart from ‘introduces and interacts with patients’, there was significant agreement for each rater across their assessments of the two different candidates, suggesting raters may have a preferred evaluation ‘style’. With respect to inter-rater reliability there was good agreement between peer-assessments across all five dimensions except ‘Social Support’ (Table 2). However, there was no agreement between peer- and self-assessments.

**Table 2:** **Inter-Rater agreements and Intra-Rater (Stability Coefficient) Effects**

	**Introduces and interacts with patient**	**Focuses on colleagues not self**	**Attends and reacts to environment**	**Avoids taking the lead**	**Offers social support**
Stability Coefficient	.17 (N =58)	.52** (N =62)	.56*** (N =66)	.26* (N =76)	.43*** (N =76)
Inter-rater agreement	.35** (N =63)	.34** (N =61)	.56*** (N =65)	.23* (N =76)	.03 (n = .73)
Self-Peer 1	.22 (N =72: p = .07)	.00 (N =68)	.03 (N =74)	.11 ( N =81)	-.06 (N =72)
Self-Peer 2	.02 (N =71)	-.03 (N =77)	-.09 (N =71)	.32** (N =75)	-.03 (N =76)

*Note*. *p < .05, **p < .01 ***P < .001.

Table 3 provides the mean scores for the peer- and self-assessments for participants where there were complete data on self-assessment, and two observer ratings^f^. A one-way between groups ANOVA shows that while self-assessments for positive behaviours (e.g., ‘Focuses on colleague rather than self’) were significantly higher than peer-assessment and lower for negative behaviours (e.g., ‘Avoids taking the lead’), there were no significant differences across the two peers’ ratings. Therefore, not only is there a significant correlation between peer-assessments but, in terms of absolute mean values, they provide identical assessments.

**Table 3:** **Mean (Standard Deviation) differences between self- and peer-assessments**

	**N per group**	**Self-assessment (S)**	**Peer-assessment (Observer 1: O1)**	**Peer-assessment (Observer 2: O2)**	***F*** **(df)**	**Contrasts**
		**Mean (SD)**	**Mean (SD)**	**Mean (SD)**		
Introduces and interacts with patient	62	7.9 (1.6)	8.8 (1.2)	8.7 (1.3)	6.7 (2,183)***	S < (O1 = O2)
Focuses on colleagues not self	60	13.3 (3.0)	15.9 (2.9)	15.7 (2.8)	15.2 (2, 177)***	S < (O1 = O2)
Attends and reacts to environment	63	10.1 (2.6)	12.5 (3.1)	12.0 (2.9)	11.8(2, 186)***	S < (O1 = O2)
Avoids taking the lead	71	6.5 (3.3)	3.0 (3.1)	3.1 (2.7)	30.6(2, 210)***	S > (O1 = O2)
Offers social support	66	1.8 (1.1)	2.1 (1.1)	2.3(1.1)	4.5(2, 195)***	S < O2, S = O1, O1 = O2

*Note*. ***p < .001. S < (O1 = O2) = Self-Assessment significantly less than Observer 1 and Observer 2, but no significant difference between Observer 1 and Observer 2. S > (O1 = O2) = Self-Assessment significantly higher than Observer 1 and Observer 2, but no significant difference between Observer 1 and Observer 2. S < O2 = Self-Assessment significantly less than Observer 2. S = O1 = no significant difference between Observer 1 and self-Assessment. O1 = O2 = no significant difference between Observer 1 and Observer 2.

### Rater-idiosyncrasy

The rater idiosyncrasy variance (%) for each dimension was: (1) 18% for ‘Focuses on colleagues rather than self’, (2) 0% for ‘Attends and reacts to the environment’, (3) 3% for ‘Avoids taking the lead’ and (4) 40% for ‘Offers social support’. In general occupation settings it has been reported that rater idiosyncrasy accounts for rating variance of 20-30%. The TRENT index rater idiosyncrasy effects were much lower than this for all categories except social support.

### Inter-correlations

The TRENT index dimensions are correlated in a theoretically consistent manner (Table 4). That is, ‘Avoids taking the lead’ was negatively associated with three of the positive behaviours: (1) ‘Focuses on colleagues rather than self’; (2) ‘Attends and reacts to the environment’ and (3) ‘Introduces and interacts with the patient’ across both peer- and self-assessments. The four positive behaviours ‘Focuses on colleagues rather than self’, ‘Attends and reacts to the environment’, ‘Offers social support’ and ‘Introduces and interacts with the patient’ were all significantly positively associated across both peer- and self-assessments.

**Table 4:** **Zero order correlations between TRENT index dimensions for self and peer ratings**

	**Introduces and interacts with patient (IPP)**	**Focuses on colleagues not self (FCNS)**	**Attends and reacts to environment A&R)**	**Avoids taking the lead (AL)**	**Offers social support (SS)**
***Self***					
IIP	1				
FCNS	.38***	1			
A&R	.43***	.65***	1		
AL	-.18	-.40***	-.44***	1	
SS	.29**	.39***	.55***	-.18	1
***Peer 1***					
IIP	1				
FCNS	.26*	1			
A&R	.42***	.67***	1		
AL	-.35**	-.49***	-.69***	1	
SS	.32**	.57***	.57***	-.40***	1
***Peer 2***					
IIP	1				
FCNS	.43***	1			
A&R	.37***	.63***	1		
AL	-.24***	-.37***	-.51***	1	
SS	.26*	.41***	.25*	-.01	1

*Note*. N =88-81 for the self rating, 76–61 for peer 1, 75–67 for peer 2 *p < .05, **p < .01 ***p < .001.

### Effect of emotions

The zero-order correlations (Table 5) between pre- and post-emotions and self-assessment confirm the prediction that the lead doctor’s pre-simulation emotions are associated with their self-assessed performance, but not the peer-assessed performance^g^ (‘Introduces and interacts with the patient’ is included for completeness although restriction of range makes reliable associations impossible). Pre-assessment negative emotions (e.g., ‘Annoyance’) were associated with reporting more instances of ‘Avoids taking the lead’. Positive pre-assessment emotions (i.e., Hedonic Tone, Energetic Arousal) were associated with increased ratings of positive behaviours (‘Focuses on colleagues rather than self’) and ameliorated against rating negative behaviours (‘Avoids taking the lead’). Importantly, post- emotions were correlated with self-assessed behaviour in the simulator. Those who report more behaviours within ‘Avoids taking the lead’ reported feeling more annoyed and higher levels of tense arousal (i.e., anxiety) and reduced both energetic arousal (i.e., active) and positive hedonic tone (i.e., happy) following the simulation.

**Table 5:** **Zero order correlations between Pre and Post emotions and Self-Reported Performance [4** ^**th**^ **order partials in parenthesis]**

	**Introduces and interacts with patient**	**Focuses on colleagues not self**	**Attends and reacts to environment**	**Avoids taking the lead**	**Offers social support**
***Pre-Mood***					
Hedonic Tone	.02	.08	-.09	-.22*	-.16
Energetic Arousal	.15	.35**	.27**	-.13	.12
Tense Arousal	.02	-.04	.05	.17	.15
Annoyed	.00	-.23*	-.01	.22*	.03
***Post-Mood***					
Hedonic Tone	.17	.35** (28*)	.26** (.33**)	-.52*** (−.48***)	.09
Energetic Arousal	.13	.39*** (.25*)	.28**	-.28** (−.27*)	.09
Tense Arousal	-.11	-.17	-.07	.37*** (.31**)	-.03
Annoyed	-.14	-.24* (−.20 *p* = .08)	-.19	.34** (.30**)	-.16

*p < .05, **p < .01, ***p < .001.

To control for the effect of pre-emotions on the association between self-assessments of performance and post-emotions, 4^th^ order partial correlations (controlling for all 4 pre-emotion levels) were calculated and are reported in Table 4 in parentheses. All effects remained significant: self-assessed performance in the simulation environment thus has a strong effect on emotions after leaving the simulation.

## Discussion

This paper contributes to the literature on the assessment of NTS in the simulated medical environment in a number of significant ways. First, the measure developed – the TRENT Index – provides for the first time a tool validated for both peer- and self-assessments. Second, the TRENT Index is based on the underlying temporal sequence common to many medical transactions. Third, it assesses a wider range of NTS (e.g., emotional reactions) than previous assessments. Fourth, the paper reports the extent to which measures of NTS are open to idiosyncrasy bias. Fifth, the TRENT was found to be internally reliable, and showed theoretically consistent correlations between scales and with pre- and post-simulation emotions. Finally, in terms of validity this paper has shown that self-assessed poor performance is associated with emotional responses both pre and post simulation. The theoretical and practical implications of these results are discussed below.

### The TRENT index: the psychometrics

The self- and peer-scales of the TRENT Index have good internal reliability. The peer-assessment demonstrated good inter-rater reliability (with the exception of ‘Offers social support’) with the size of the correlations equivalent to those reported in the wider literature on peer-assessments. The assessment of social support had a strong *idiosyncrasy* bias, and as such the assessment of social support reflects an idiosyncratic view, which should be acknowledged when used in practice. The extent to which this type of bias is present in currently used peer assessments (e.g., anaethetists’ non-technical skills: ANTS) is, therefore, a concern that needs to be explored further.

Whilst there was evidence for inter-rater reliability there was no evidence for agreement between self- and peer-assessment. This does not mean that the TRENT index lacks validity, rather it means that peer- and self-assessments provide different information about the observed doctor. Once this is acknowledged and the relative biases in each are known, the two sources of information can be combined to provide more comprehensive NTS assessment and feedback. For example, any discrepancy between the self- and peer-assessments can be used as a point for discussion with Clinical or Educational Supervisors and used to question not only how the doctors view themselves, but also how they are viewed by others. Also, both peer- and self-assessed performance clearly relate to different aspects of the simulation experience, as the links between performance and emotion (see below) show. Finally, the correlations between the TRENT scales and emotions indicated good construct validity.

With respect to generalizability we can ask the question whether the TRENT Index can be used in hospital ward contexts (emergency and elective) outside the simulated environment. While this ultimately is an empirical question (and one that deserves further research) we feel that the domains of NTS assessed by TRENT index and the temporal order that underlies its makes it generalizable to the actual work place.

### The role of emotions in medical simulation and assessment

The associations reported between emotions and self- and peer-assessed performance highlight important issues pertaining to (1) the validity of both self- and peer-assessment and (2) potential negative impacts of simulation training on the transfer of learning.

In terms of validity, and consistent with the theory [30,31], doctors’ emotions prior to entering the simulation influenced how they rate their own performance [30,31], but the observers’ assessment of the doctor’s performance was unrelated to the observed doctor’s emotions. As there was no association between peer-assessed performance and the assessed doctor’s pre- or post-simulation emotions, peer-assessments may not take into account how the assessed doctor’s emotions relate to their performance [30,31]. Thus peer-assessed performance may not be able to identify how psychological processes, such as emotions, influence performance. This again emphasises why having both self- and peer-assessments is crucial and why peer assessments cannot necessarily be seen as the ‘gold standard’.

The results showed that those who were anxious, self-rated their behaviour as more negative and less positive. The converse is true for those expressing positive emotions (i.e., happy & relaxed). Thus self-assessment is influenced by the emotions of the performing doctor, in a way that peer-assessment of performance is not. Similarly, in real ward contexts the doctor is likely to have emotional experiences prior to meeting a patient and this may affect their interactions and decision-making. These results highlight that the simulated medical context does influence emotions and performance akin to the real ward context [38,39]. If only peer-assessed performance had been assessed, this may lead to the conclusion that emotions and performance are not linked in this context, which is not the case. Future studies should, however, examine the emotions of the peer assessor to explore if they influence their peer-assessments of others. Both self- and peer-assessments are, therefore, crucial as they assess different aspects of performance, challenging the view of peer-assessment as the gold standard.

In terms of transfer of training, the results showed, for the first time, that self-assessed poor performance in the simulator is associated with increased negative emotions following the simulation. This has important implications for the clinical utility of simulation and the transfer of training back to the work context. If the doctors leave the simulation in a negative mood, this may have the potential to generalize beyond the simulated environment, influencing their performance when they return to work. To date there is no evidence on how emotion in the simulated medical context generalizes back to the work context, but the results reported here suggest that this might be an issue that needs to be considered. It also suggests that more is needed to be known about factors affecting post-simulation emotions, such as role (e.g., physician versus nurse) [17].

While the TRENT index can be used by trainees, we believe it should also be used by expert faculty staff to rate the doctors in each ongoing scenario. Thus a line of future research would be to extend the use of TRENT index to expert faculty staff.

The results reported here extend previous work showing that simulated medical contexts are stressful [28,29], by showing that links between stress (negative emotions) generated before and after being in a simulated medical environment is associated with performance in the simulator.

## Conclusions

The paper provides evidence on the robust psychometric qualities of the newly developed TRENT Index which provides, for the first time, both peer- and self-assessments of non-technical skills (NTS). The paper reports the extent to which measures of NTS such as the TRENT Index are open to idiosyncrasy biases. Finally the paper shows that self-assessed poor performance in a high fidelity acute care simulation is associated with negative emotional responses both pre- and post-simulation. This suggests that negative emotions can arise during acute care simulation, and may affect later performance.

## Endnotes

^a^Foundation Programme doctors in the UK are those who have just graduated their undergraduate medical degrees and then spend 2 years (Foundation years 1 and 2 called F1 and F2 respectively) gaining relevant experience to become finally registered as a Doctor. (see for more details: http://www.nhscareers.nhs.uk/explore-by-career/doctors/training-to-become-a-doctor/foundation-training/).

^b^Of the originally 27 items, one item was problematic due to being identified as singular in the analyses.

^c^A good fitting model has a Comparative Fit Index (CFI) approaching .96, a Root Mean Square Error of Approximation (RMSEA) approaching .06 and a Weighted Root Mean Square Residual (WRMR) of 1 or less [40].

^d^Full details regarding these analyses in terms of treating missing data, item deletion and cross-loading suggested by modification indices are available from the first author on request.

^e^The second doctor also provided pre- and post-assessments of their emotions but these are not analysed in this paper.

^f^When the same analyses were conducted on all the data, which included missing data on one or more type of assessment, the pattern of results was identical.

^g^The peer-assessments associations are not reported as there were too few (4 of 80) significant associations to signify any systematic effect.

## Additional file

Additional file 1:
**TRENT index Form and Psychometrics.**
