# Supplementary material for: The Temporal Rating of Emergency Non-Technical skills (TRENT) index for self and others: psychometric properties and emotional responses
Source: BMC Med Educ. 2014 Nov 28;14:240. doi: 10.1186/s12909-014-0240-y (PMC4330940; doi:10.1186/s12909-014-0240-y)
Supplement: Additional file 1: — TRENT index Form and Psychometrics. [file 12909_2014_240_MOESM1_ESM.doc]

**Additional file 1:** TRENT index Form and Psychometrics

|  |  | Target’s perception of | | Peer#1 perception of | Peer#2 perception of |
| --- | --- | --- | --- | --- | --- |
|  | Self | Peer # 1 | Peer # 2 | Target | Target |
| **Introduces and interacts with the patient** | α = .67 | α = .14 | α = .59 | α = .46 | α = .34 |
| Introduces self |  |  |  |  |  |
| Uses patients name |  |  |  |  |  |
| Listens to patient and/or relative |  |  |  |  |  |
| Takes time to explain to patient what’s happening |  |  |  |  |  |
| Builds a good rapport with patient |  |  |  |  |  |
| **Focuses on colleagues rather than self** | α = .74 | α = .76 | α = .74 | α = .77 | α = .72 |
| Uses colleagues names |  |  |  |  |  |
| Makes plans and instructions clear so that colleagues should know what they are being asked to do |  |  |  |  |  |
| Involves colleagues in decision making |  |  |  |  |  |
| Listens to colleagues |  |  |  |  |  |
| Is reactive to colleagues instructions |  |  |  |  |  |
| Communicates plans to colleagues |  |  |  |  |  |
| Takes the initiative |  |  |  |  |  |
| Checks that colleagues understand what is required |  |  |  |  |  |
| Works on their own when help or assistance from colleagues would be beneficial (*reverse scored*) |  |  |  |  |  |
| Empowers colleagues to contribute |  |  |  |  |  |
| **Attends and reacts to the environment** | α = .78 | α = .79 | α = .81 | α = .83 | α = .76 |
| Reacts to changes in circumstances appropriately |  |  |  |  |  |
| Pays attention to circumstances around them |  |  |  |  |  |
| Quickly and correctly prioritises on the basis of clinical need and test results |  |  |  |  |  |
| Orders inappropriate tests or equipment (*reverse scored*) |  |  |  |  |  |
| Orders appropriate tests or equipment |  |  |  |  |  |
| Orders tests or equipment in good time |  |  |  |  |  |
| Seeks appropriate advice or help |  |  |  |  |  |
| Seeks appropriate advice or help as soon as it is needed |  |  |  |  |  |
| **Avoids taking the lead** | α = .82 | α = .86 | α = .75 | α = .86 | α = .74 |
| Becomes withdrawn during a crisis |  |  |  |  |  |
| Is apprehensive when making decisions |  |  |  |  |  |
| Approaches problems with confidence (*reverse scored*) |  |  |  |  |  |
| Avoids taking the lead |  |  |  |  |  |
| Tackles problems head on (*reverse scored*) |  |  |  |  |  |
| Starts to become anxious |  |  |  |  |  |
| Remains calm (*reverse scored*) |  |  |  |  |  |
| Waits for someone else to prioritise |  |  |  |  |  |
| **Offers social support** | MIC = .56 | MIC = .31 | MIC = .50 | MIC = .49 | MIC = .35 |
| Offers *emotional* support to colleagues when appropriate | α = .73 | α = .47 | α = .66 | α = .66 | α = .51 |
| Offers *practical* support to colleagues when appropriate |  |  |  |  |  |
